# Rapid and Low-Cost Culture-Based Method for Diagnosis of Mucormycosis Using a Mouse Model

**DOI:** 10.3389/fmicb.2020.00440

**Published:** 2020-03-20

**Authors:** Afsane Vaezi, Hamed Fakhim, Macit Ilkit, Leila Faeli, Mahdi Fakhar, Vahid Alinejad, Nathan P. Wiederhold, Hamid Badali

**Affiliations:** ^1^Invasive Fungi Research Center, School of Medicine, Mazandaran University of Medical Sciences, Sari, Iran; ^2^Department of Medical Mycology, School of Medicine, Mazandaran University of Medical Sciences, Sari, Iran; ^3^Department of Medical Parasitology and Mycology, Faculty of Medicine, Urmia University of Medical Sciences, Urmia, Iran; ^4^Infectious Diseases and Tropical Medicine Research Center, Isfahan University of Medical Sciences, Isfahan, Iran; ^5^Division of Mycology, Department of Microbiology, Faculty of Medicine, University of Çukurova, Adana, Turkey; ^6^Student Research Committee, Mazandaran University of Medical Sciences, Sari, Iran; ^7^Toxoplasmosis Research Center, Department of Parasitology, Mazandaran University of Medical Sciences, Sari, Iran; ^8^Patient Safety Research Center, Urmia University of Medical Sciences, Urmia, Iran; ^9^Fungus Testing Laboratory, Department of Pathology and Laboratory Medicine, University of Texas Health Science Center at San Antonio, San Antonio, TX, United States

**Keywords:** microculture, rapid diagnosis, *Rhizopus arrhizus*, mucormycosis, murine model

## Abstract

Prompt and targeted antifungal treatment has a positive impact on the clinical outcome of mucormycosis; however, current diagnostic tools used in histopathology laboratories often fail to provide rapid results. Rapid culture-based strategies for early diagnosis of *Mucorales* infections, which may influence treatment decisions, are urgently needed. Herein, we evaluated a microculture assay for the early diagnosis of mucormycosis in an immunocompetent murine model of disseminated infection, by comparing it with traditional diagnostic methods. The assay specificity was assessed using blood (*n* = 90) and tissue (*n* = 90) specimens obtained from mice infected with *Rhizopus arrhizus* using different inoculum sizes [1 × 10^4^, 1 × 10^5^, and 1 × 10^6^ colony forming units (CFUs)/mouse] and blood (*n* = 15) and tissue specimens (*n* = 15) from uninfected mice. Surprisingly, 26 of 90 (28.9%) blood samples revealed positive results by microculture, whereas all blood samples were negative when assayed by conventional culture. The overall positive conventional culture rate for the mouse tissue (kidney) samples was 31.1% (28/90). The calculated sensitivity for kidney microculture was 98.8% [95% confidence interval (CI) 96.6–100], with an assay specificity of 100%. Hence, the microculture assay may be useful for rapid culturing and diagnosis of mucormycosis caused by *R. arrhizus* directly in blood and tissue samples. Hence, this method may allow for the timely administration of an appropriate treatment.

## Introduction

Mucormycosis (previously called zygomycosis), an aggressive infection caused by mucoralean fungi, is the third most prevalent fungal disease after candidiasis and aspergillosis, among populations at high risk, including those with uncontrolled diabetes, solid organ or allogeneic stem cell transplant recipients, and patients undergoing immunosuppressive therapies (Petrikkos et al., 2012; Danion et al., 2015; Farmakiotis and Kontoyiannis, 2016; Hoenigl et al., 2018; Cornely et al., 2019). The reported incidence of mucormycosis is 0.2–95 cases per 1,000,000 individuals in Europe; 3.0 cases per 1,000,000 individuals in the United States; 1.2 cases per 1,000,000 individuals in Canada; and 0.6 cases per 1,000,000 individuals in Australia (Skiada et al., 2012). Although few studies in Asia have reported the prevalence of *Mucorales* infections (Yamazaki et al., 1999; Chakrabarti and Singh, 2014; Vaezi et al., 2016), a national investigation of medical autopsies revealed that the incidence of these infections in Japan increased by 16% in a 20-year span (Prakash and Chakrabarti, 2019). Early diagnosis and application of multimodal treatment, including appropriate antifungal therapy, may have a positive impact on clinical outcomes in patients with mucormycosis, including improved survival rates (Spellberg et al., 2005; Skiada et al., 2018).

The current gold standard for diagnosing mucormycosis is based on histopathological and mycological findings, followed by unspecific radiological criteria. However, these procedures require specialized expertise and the results are often not available in a timely fashion (Hammond et al., 2011; Cornely et al., 2019). To enhance outcomes, patients suspected to have mucormycosis should be immediately treated. Despite our improved understanding of the disease and the availability of various medico-surgical treatments, the survival rate in mucormycosis patients remains poor (Kontoyiannis and Lewis, 2011; Katragkou et al., 2014; Pilmis et al., 2018; Skiada et al., 2018). Therefore, there is a need for novel diagnostic assays. Several new molecular methods for the diagnosis of mucormycosis have been reported (Hammond et al., 2011; Dadwal and Kontoyiannis, 2018); however, these techniques may lack sensitivity, can be time-consuming and expensive to perform, and are not universally available (Katragkou et al., 2014).

The choice of an effective treatment regimen against mucormycosis requires early diagnosis and identification of the causative pathogen and its antifungal susceptibility profile, for which a positive culture is needed (Walsh et al., 2014; Hoenigl et al., 2018; Cornely et al., 2019). However, due to the unique physiology of these etiological agents (e.g., fragile and non-septate hyphae), cultures are frequently negative, and the processing of clinical specimens requires a suspicion of *Mucorales* as the causative agent and experienced laboratory personnel than may be required for fungi with septate hyphae (Ben-Ami et al., 2009; Hammond et al., 2011; Lewis et al., 2013; Cornely et al., 2019).

Here, we report the first study to evaluate a microculture assay as a putative, rapid, and low-cost culture-based method for the early diagnosis of mucormycosis. An established murine model was utilized to compare the performance of this microculture assay with those of traditional diagnostic methods.

## Materials and Methods

### Isolate and Inocula

*Rhizopus arrhizus* var. *arrhizus* clinical isolate (CBS 112.07), obtained from the reference culture collection of the Westerdijk Fungal Biodiversity Institute (Utrecht, Netherlands), was used in this study. Species identity was confirmed by DNA sequence analysis of the internal transcribed spacer (ITS) region of ribosomal DNA (rDNA), as previously described (Nagao et al., 2005). For inoculum preparation, the strain was sub-cultured onto potato dextrose agar (PDA) at 37°C (Difco, Leeuwarden, Netherlands) 10 days before the inoculation in mice to ensure viability and purity. On the day of inoculation, sterile phosphate-buffered saline (PBS) containing 0.1% (v/v) Tween 20 was added to the plate, and the surface of colonies was gently scraped. After centrifugation at 15,000 rpm for 15 min, the supernatant was removed, and the cells were washed twice in PBS. The spore count was enumerated with a hemocytometer for preparing the final inocula. The cell concentrations were adjusted to three different inoculum sizes, 1 × 10^4^, 1 × 10^5^, and 1 × 10^6^ spores/ml. To confirm each inoculum size, dilutions were prepared and streaked onto PDA, and the fungal colonies were enumerated after 24 h of incubation at 30°C.

### Animal Model

Female immunocompetent ICR mice (weighing 22–25 g, 6-week old, *n* = 105) were purchased from the Royan Institute (Tehran, Iran). The animals were housed in groups of 30 mice each at the Animal Experimentation Facility under standard conditions. All mice were provided food and water *ad libitum* and were monitored daily, based on the recommendations of the guide for the Care and Use of Laboratory Animals of the National Research Council (2011). All animal experiments were approved by the Institutional Animal Ethical Committee (IAEC) of Mazandaran University of Medical Sciences, Sari, Iran (IR.MAZUMS.REC.1397.9).

### Experimental Model of Disseminated Infection

In total, 90 mice were randomly divided into three groups (*n* = 30 per group), and a 0.2 ml solution containing one of the three inocula [1 × 10^4^, 1 × 10^5^, and 1 × 10^6^ colony forming units (CFUs)/mouse] was injected into the lateral tail vein of each mouse. Pilot experiments demonstrated that the inoculum sizes of 1 × 10^4^, 1 × 10^5^, and 1 × 10^6^ CFU/mouse proved to be the optimal doses leading to a severe infection; all animals died within 10 days of infection. In the fungal burden arm, mice were sacrificed by cervical dislocation on experimental day 4 post-infection. After sacrifice, kidneys were removed, homogenized, serially diluted (1:10), and plated on Sabouraud dextrose agar (SDA) for CFU/g calculation. The control group (*n* = 15) received intravenous injections of cell-free PBS using the same method. Mice were assessed at least twice daily, and moribund mice were euthanized by exsanguination (intracardiac puncture under general anesthesia) (Conti et al., 2014) after detecting symptoms of disseminated infection. Moribund animals were identified by the following criteria: decreased activity, inability to eat or drink, hypothermia, hunched posture, and torticollis or barrel rolling.

### Histopathological and Mycological Characterization

Blood and tissue (kidney) samples were recovered under aseptic conditions. Blood samples were collected by cardiac puncture (approximately 200 μl into heparin-coated tubes) and were stored at −20°C until further analysis. The kidneys were minced and used for histopathology, microculture assay, traditional culture analysis. Kidney samples were first fixed in 10% (w/v) buffered formalin, dehydrated, paraffin-embedded (FFPE), sectioned (5-μm-thick sections), and stained with Periodic acid–Schiff (PAS) for direct microscopic examination, as previously described (Rickerts, 2016). Minced kidney and blood samples were also cultured on SDA and brain heart infusion (BHI) agar at 35°C. Moreover, the blood samples were inoculated into diphasic blood-culture bottles containing BHI broth and agar (Kusha Faravar Giti, Karaj, Iran). The samples were incubated at 37°C for at least 2 weeks. The remainder of the kidney samples and additional blood samples were used for microculture assay. Furthermore, control kidney and blood samples were also collected from uninfected mice for analysis.

### Microculture Assay

For the assay, 50 μl of blood was sampled using a sterile non-heparinized 1 × 75 mm glass capillary tube. Blood sampling was performed by extracting the blood directly into the capillary tubes. For tissue samples, 20–50 mg of minced kidney was inserted into a sterile glass Pasteur pipette (146 × 6.5 mm). The capillary tubes and Pasteur pipette were then loaded with 50–70 μl and approximately 200 μl of RPMI 1640 medium (Sigma, St. Louis, MO, United States), respectively. The tubes were sealed with wax and incubated at 35°C. One capillary tube sample and one Pasteur pipette sample were prepared for each animal. All samples were examined daily using an inverted microscope (Motic AE31 Elite Inverted Phase Contrast Microscope, magnification 100×). Culture-negative samples were monitored for up to 30 days. Capillary tubes were examined under a light microscope (Nikon YS100 Biological Microscope, magnification 400×). For the light microscopy analysis, two capillary tubes were placed horizontally on a microscope slide, and another slide was placed over them. The gap between the slides was filled with sterile water.

### Statistical Analysis

Median survival time was estimated by the Kaplan–Meier method, compared among groups by the log-rank test. Tissue burden data of tested organ in the different experimental groups were analyzed by using the Kruskal–Wallis test in SPSS (version 17.0 for Windows; Chicago, IL, United States) and plotted using GraphPad Prism version 6.01 (Graph Prism Software Inc., United States). Categorical differences between positive and negative results for samples between the traditional culture (SDA) and microculture techniques were also determined by the equality of mean differences using a Chi-square 2 × 2 contingency table at a 95% confidence interval. *p-*values < 0.05 were considered as statistically significant.

## Results

**FIGURE 1:**
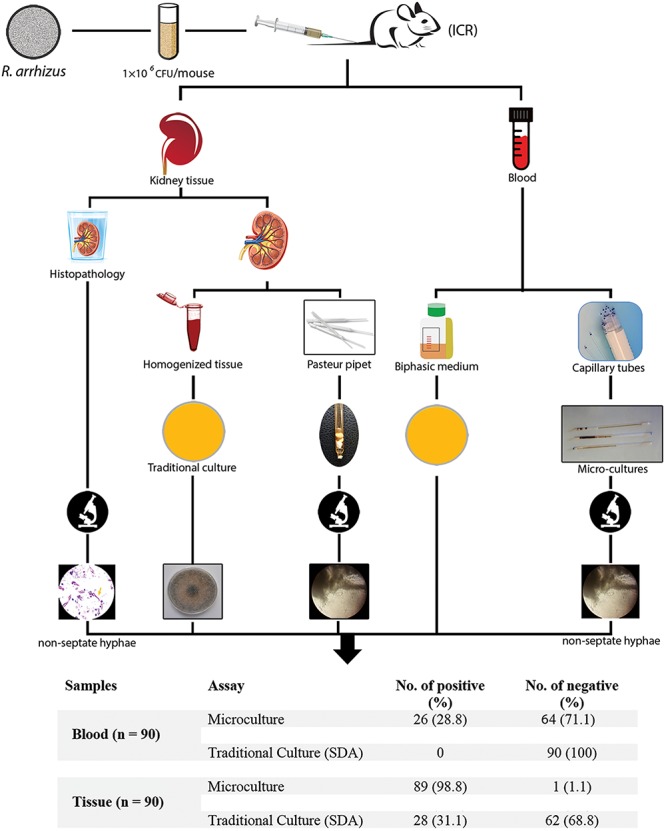
Schematic of this study evaluating microculture compared with the traditional diagnostic methods.

An overall schematic of this study of the evaluation of microculture for the early diagnosis of mucormycosis in an immunocompetent murine model of disseminated infection compared with routinely performed methods is shown in Figure 1.

**FIGURE 2:**
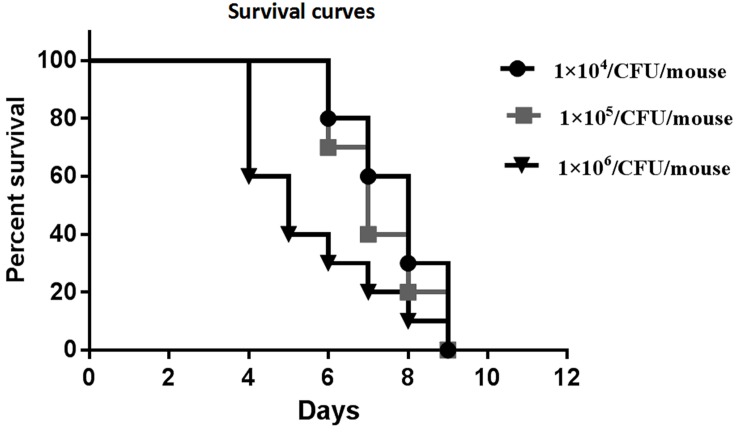
Survival curves of preliminary experiments carried out using three different inoculum sizes [1 × 10^4^, 1 × 10^5^, and 1 × 10^6^ colony forming units (CFUs)/mouse] for each group, which consisted of 10 mice intravenously (IV) infected with the inocula of the *Rhizopus arrhizus* clinical isolate.

**FIGURE 3:**
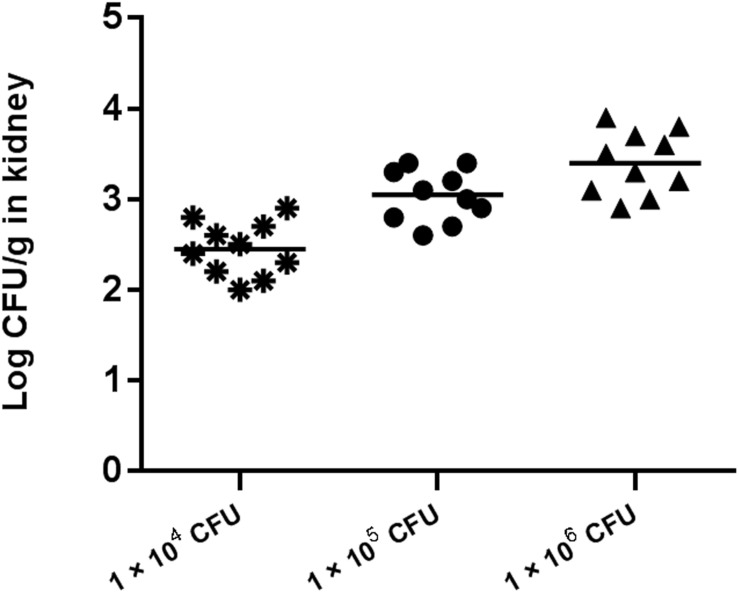
Fungal tissue burden results in ICR mice infected with inocula of 1 × 10^4^, 1 × 10^5^, and 1 × 10^6^ CFU/mouse on day 4.

Preliminary experiments using three different inocula demonstrated that median survival time was 8, 7, and 5 days for mouse inoculation with 1 × 10^4^, 1 × 10^5^, and 1 × 10^6^ CFU/mouse, respectively (Figure 2). The fungal tissue burden results are summarized in Figure 3. Fungal burden was significantly higher in mice infected with 1 × 10^6^ CFUs compared to the other inocula levels in the 1 × 10^4^ and 1 × 10^5^ CFU/mouse groups (*p* < 0.001).

**FIGURE 4:**
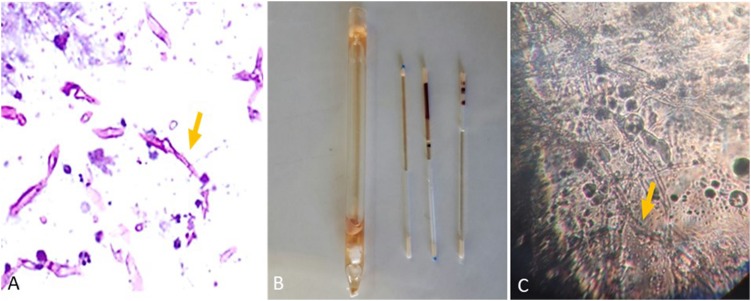
**(A)** Representative histopathological periodic acid-Schiff (PAS) section of kidney from Institute of Cancer Research (ICR) female mice intravenously infected with *R. arrhizus* (1 × 10^6 ^ CFU/mouse) tested on day 7 post-challenge. **(B)** Blood and tissue sampling for the microculture assay using a sterile non-heparinized glass capillary tube. **(C)** Images captured 18 h after commencing the experiment (*Mucorales hyphae*).

Histopathological examination of kidney sections stained with PAS revealed irregular, broad, and non-septate hyphae, hallmarks of *Mucorales*, with several foreign bodies and langhans giant cells surrounded by a dense inflammatory response (Figure 4A). Subsequently, the performance of the microculture assay was evaluated by comparing it with culturing on media plates.

### Diagnostic Performance of the Microculture Assay

#### Blood Samples

The animals were euthanized on days 3–7 post-infection. Of the 90 blood samples, 26 (28.9%) were positive using the microculture assay (Figures 4B–C). This included 6 in the lower inoculum group, 11 in the medium inoculum group, and 9 in the high inoculum group. Microculture samples in each group were positive between days 3 and 7 post-inoculations. In contrast, all blood samples were negative by culture (on SDA and BHI).

#### Kidney Samples

Microculture results were positive in 89 of 90 kidney samples (98.9%). The overall positive conventional culture rate for the mouse kidney samples was 31.1% (28/90). Therefore, 89 samples presented a positive result for kidney microculture assay, of which 28 were in accordance with the results of the SDA culture. There was significant difference in the positive microculture compared with SDA plates (*p* < 0.0001). All the samples collected from the 15 uninfected mice were negative by each assay. The concordance of detection (calculated sensitivity) for kidney microculture was 98.8% (95% CI 96.6–100), with a calculated assay specificity of 100%. For kidney samples, the SDA culture presented the lower sensitivity (33.7%; 95% CI 23.9–43.5). These data demonstrate that the microculture assay (98.9% positivity) is superior to conventional culture (31.1% positivity) in this murine model in detecting *R. arrhizus* directly from the primary blood and kidney samples within 18–24 h of sampling.

## Discussion

In this study, we present the results of a microculture assay for timely culture of *R. arrhizus.* This is the first study to demonstrate that a microculture approach can be used for fungal culture within 24 h of sampling. Early diagnosis via multiple approaches is an important aspect of care in patients with mucormycosis (Blyth et al., 2011; Walsh et al., 2014; Hoenigl et al., 2018). A timely and efficient diagnosis, as well as an aggressive multimodal treatment approach, is critical in the management of this fulminant progressive and invasive disease, as delays may result in an increased mortality risk (Walsh et al., 2014; Jeong et al., 2016). Indeed, a delay of more than 5 days of an effective antifungal therapy in patients with hematological malignancies leads to approximately twofold increase in 12-week mortality (Spellberg et al., 2005; Chamilos et al., 2008; Palejwala et al., 2016). Rapid mycological diagnostic methods may assist with timely initiation of appropriate antifungal therapy, which may prevent progressive tissue invasion, lead to decreased mortality, and overall improvement in healthcare utilization (i.e., shorter hospitalization duration and reduced costs). Histological analysis is an important diagnostic tool in the early management of this devastating disease (Skiada et al., 2018); however, the 24-h turnaround time of the microculture approach is considerably shorter than that of histological analysis (48–72 h) (Dekio et al., 2015) or conventional culturing (3–7 days) (Skiada et al., 2018).

Conventional tissue fungal cultures are typically positive in only 50% of mucormycosis cases (Skiada et al., 2018). Positive cultures and fungal identification, even at the genus level only, allow for the appropriate choice of antifungal regimens and further assessment of antifungal resistance patterns and emerging resistance (Cornely et al., 2014; Beardsley et al., 2018). Although some molecular identification methods may be able to provide results within a few hours, the microculture assay described in this study does not require specialized training or equipment. Surprisingly, in the present study, 26 of 90 blood samples were positive by microculture, while all blood samples were negative with traditional culture. Increased CO_2_ levels during incubation, leading to a lower pH, may facilitate the growth of *R*. *arrhizus* in microculture tubes.

Culture-based methods for fungal identification are generally practical, economical, and accessible. The microculture method presented in this study is relatively rapid and easy to perform. Hence, this approach could also be considered for the diagnosis of other fungal infections, which may be challenging using traditional culture methods.

The promising results of the present study require confirmation through further studies. Microculture methods should be further assessed for the detection of other members of the order *Mucorales* as well as in other fungi. In addition, the performance of this assay in other murine models (e.g., pulmonary mucormycosis and in immunosuppressed hosts) should be evaluated. These additional studies are warranted given the relative ease of use of this method and the impact it may have within the clinical microbiology laboratory.

## Author’s Note

A part of this work was presented as a poster presentation at the 20th Congress of the International Society for Human and Animal Mycology (ISHAM), Amsterdam, 30 June to 4 July 2018.

## Data Availability Statement

The raw data supporting the conclusions of this article will be made available by the authors, without undue reservation, to any qualified researcher.

## Ethics Statement

This study was carried out in accordance with the recommendations of Guide for the Care and Use of Laboratory Animals, Committee for the Update of the Guide for the Care and Use of Laboratory Animals. The protocol was approved by the Ethics and Research Committee of Mazandaran University of Medical Sciences, Sari, Iran (IR.MAZUMS.REC.1397.9).

## Author Contributions

AV and HB contributed to the design and implementation of the research, and drafted the manuscript. AV, HB, and HF curated the data. AV, HB, and VA performed the formal analysis of the study and contributed to funding acquisition and project administration. AV, MF, HF, and LF provided the methodology for this study. MI and NW validated the data and revised the manuscript. All authors contributed to approve the final version of the manuscript.

## Conflict of Interest

The authors declare that the research was conducted in the absence of any commercial or financial relationships that could be construed as a potential conflict of interest.

The reviewer AA-H declared past co-authorship with several of the authors, AV, HF, MI, and HB, to the handling Editor.
